# Application of DNA adductomics to soil bacterium *Sphingobium* sp. strain KK22

**DOI:** 10.1002/mbo3.283

**Published:** 2015-08-25

**Authors:** Robert A Kanaly, Ruggero Micheletto, Tomonari Matsuda, Youko Utsuno, Yasuhiro Ozeki, Natsuko Hamamura

**Affiliations:** 1 Department of Life and Environmental System Science, Graduate School of Nanobiosciences, Yokohama City UniversityKanagawa, Yokohama, 236-0027, Japan; 2 Department of Nanosystem Science, Graduate School of Nanobiosciences, Yokohama City UniversityKanagawa, Yokohama, 236-0027, Japan; 3 Research Center for Environmental Quality Management, Kyoto UniversityOtsu, Shiga, 520-0811, Japan; 4 Center for Marine Environmental Studies, Ehime UniversityMatsuyama, 790-8577, Japan; 5 Department of Biology, Faculty of Sciences, Kyushu UniversityFukuoka, 812-8581, Japan

**Keywords:** Acrolein, bacterium, DNA adductomics, DNA adducts, LC-MS/MS, *Sphingobium*

## Abstract

Toward the development of ecotoxicology methods to investigate microbial markers of impacts of hydrocarbon processing activities, DNA adductomic analyses were conducted on a sphingomonad soil bacterium. From growing cells that were exposed or unexposed to acrolein, a commonly used biocide in hydraulic fracturing processes, DNA was extracted, digested to 2′-deoxynucleosides and analyzed by liquid chromatography-positive ionization electrospray-tandem mass spectrometry in selected reaction monitoring mode transmitting the [M + H]^+^ > [M + H − 116]^+^ transition over 100 transitions. Overall data shown as DNA adductome maps revealed numerous putative DNA adducts under both conditions with some occurring specifically for each condition. Adductomic analyses of triplicate samples indicated that elevated levels of some targeted putative adducts occurred in exposed cells. Two exposure-specific adducts were identified in exposed cells as 3-(2′-deoxyribosyl)-5,6,7,8-tetrahydro-6-hydroxy-(and 8-hydroxy-)pyrimido[1,2-*a*]- purine-(3*H*)-one (6- and 8-hydroxy-PdG) following synthesis of authentic standards of these compounds and subsequent analyses. A time course experiment showed that 6- and 8-hydroxy-PdG were detected in bacterial DNA within 30 min of acrolein exposure but were not detected in unexposed cells. This work demonstrated the first application of DNA adductomics to examine DNA damage in a bacterium and sets a foundation for future work.

## Introduction

DNA adductomics is an emerging field that was conceived of as a top down comprehensive nontargeted approach to detect DNA adducts in human tissues by utilizing liquid chromatography coupled with electrospray ionization tandem mass spectrometry (LC/ESI-MS/MS; Kanaly et al. 2006, 2007; recently reviewed by Balbo et al. 2014b). LC/ESI-MS/MS is now established as a powerful technique for the analysis of DNA adducts owing to its high sensitivity and selectivity (Singh and Farmer 2006; Tretyakova et al. 2013) and DNA adductomics strives to maximize these parameters while at the same time aims to overcome the challenges of screening for a wide range of unknown analytes.

By using the DNA adductome approach, known DNA adducts may be detected, while at the same time yet uncharacterized DNA adducts may be revealed that would not have been discovered if only targeted approaches were employed. Specifically, the DNA adductomics strategy utilizes a triple quadrupole MS to detect the neutral loss of 2′-deoxyribose from positively ionized 2′-deoxynucleoside adducts by using single reaction monitoring (SRM) mode transmitting the [M + H]^+^ > [M + H − 116]^+^ transition over a range of multiple predefined transitions (Kanaly et al. 2007; Chou et al. 2010; Matsuda et al. 2013). When putative DNA adducts that are revealed by DNA adductomics are targeted for tandem mass product ion scan analyses, the screening power of the technique may be extended. Finally, to confirm the identities of putative DNA adducts, comparative analyses with authentic DNA adduct standards is necessary.

Until now, DNA adductomics has been applied to humans, mice and food (Kanaly et al. 2006, 2007; Bessette et al. 2008; Chou et al. 2010; Singh et al. 2010; Spilsberg et al. 2010; Matsuda et al. 2013; Balbo et al. 2014a; Ishii et al. 2014), however, DNA modifications in microorganisms may also serve as potential markers of exposure to toxic pollutants (Gaskell et al. 2004; Szyf 2011). Sphingomonad bacteria are known to occur at sites contaminated with hazardous hydrocarbon pollutants and they may serve as representative organisms from such sites because they can maintain their existence under polluted conditions (Cunliffe and Kertesz 2006; Kertesz and Kawasaki 2010). As part of the development of methods to investigate microbial markers of impacts of hydrocarbon processing activities such as hydraulic fracturing on the environment, a sphingomonad soil bacterium that was capable of withstanding concentrations of potentially genotoxic organic hydrocarbon pollutants such as benz[*a*]anthracene and fluoranthene in the 100 ppm range was selected as a model organism for this investigation (Kunihiro et al. 2013; Maeda et al. 2014). For comparison, the maximum contaminant level for benz[*a*]anthracene in drinking water is 1 million times less (US EPA 2000). At the same time, the small molecule, acrolein, which is used in environmental applications where it is applied as a biocide in hydraulic fracturing processes and oilfield systems was chosen as a model toxicant (Hemminki et al. 1980; Foiles et al. 1989; Nordone et al. 1996; Penkala et al. 2004).

In this investigation, multiple DNA adductomics analyses were conducted on growing *Sphingobium* cells, with the following aims: (1) to evaluate potential differences in DNA damage depending upon exposure conditions, (2) to unambiguously identify unknown adducts that may have occurred due to exposure to acrolein from complex data sets, and (3) to build a foundation upon which future prokaryotic DNA adductomics studies may be conducted.

## Experimental Procedures

### Biochemical and chemical reagents

Micrococcal nuclease (MN) and bovine spleen phosphodiesterase II (SPD) were purchased from Worthington Biochemical Corp. (Lakewood, NJ). Bacterial alkaline phosphatase Type III (*Escherichia coli*), 2′-deoxycytidine, 2′-deoxythymidine, 2′-deoxyguanosine, 2′-deoxyadenosine, 2′,3′-dideoxyinosine, and phenanthrene were purchased from Sigma-Aldrich Co. (St. Louis, MO). Prop-2-enal, acrolein, was purchased from Tokyo Chemical Industries (Tokyo, Japan). Methanol, ethanol, diethyl ether (all LC grade or higher), dimethyl sulfoxide (DMSO) and glucose were purchased from Wako Chemical (Osaka, Japan).

Analytical standards of modified 2′-deoxynucleosides, 1,*N*^6^-etheno-2′-deoxyadenosine, and 8-hydroxy-2′-deoxyguanosine were purchased from Sigma-Aldrich. *N*^6^-methyl-2′-deoxyadenosine was purchased from Berry and Associates, Inc. (Dexter, MI). 2′-deoxy-5-methylcytidine was purchased from Tokyo Chemical Industries (Tokyo, Japan). *N*^*2*^-ethyl-2′-deoxyguanosine, 3-(2′-deoxyribosyl)-5,6,7,8-tetrahydro-8-hydroxy-pyrimido[1,2-*a*]purine-(3*H*)-one (8-hydroxy-PdG), and the two stereoisomers 3-(2′-deoxyribosyl)-5,6,7,8-tetrahydro-6-hydroxypyrimido [1,2-*a*]purine-(3*H*)-one (6-hydroxy-PdG) were synthesized similarly to as described previously (Kanaly et al. 2007). In the case of 6- and 8-hydroxy-PdGs, 3 mg of 2′-deoxyguanosine was reacted with 5 *μ*L of acrolein in 1.2 mL of pH 7.0 phosphate buffer for 48 h in the dark in a 37°C water bath with reciprocal shaking at 150 rpm. The resulting reaction mixture was purified by reverse phase column chromatography with Sep-Pak C18 cartridges according to the manufacturer's instructions (Waters Corp., Milford, MA). Solutions were concentrated in vacuo, resuspended in DMSO and concentrated by LC column chromatography by using a Waters 2690 Separations Module (Waters, Corp., Milford, MA) that was in-line with a Waters 2998 photodiode array detector. Reaction products were applied to a Shim-pack XR-ODS column (3.0 × 75 mm, Shimadzu, Kyoto, Japan). The mobile phase consisted of a methanol and water gradient (5% methanol) that transitioned to 80% methanol over a period of 25 min at a flow rate of 0.2 mL/min.

### Bacterial strain

*Sphingobium* sp. strain KK22 was isolated from a soil microbial consortium that was studied for its ability to grow on diesel fuel and biotransform polycyclic aromatic hydrocarbons (PAHs) (Kanaly et al. 1997, 2000). The catabolic capabilities of this strain were recently reported (Kunihiro et al. 2013; Maeda et al. 2014) and it was characterized (Maeda et al. 2015). Strain KK22 was maintained on 300 mg L^−1^ phenanthrene in Stanier's Basal Medium (SBM) (Atlas 1993) as a sole source of carbon and energy by continuous rotary shaking at 150 rpm at 30°C in the dark.

### Growth and exposure conditions

In all experiments, strain KK22 cells were cultivated on 20 mmol/L glucose in SBM to mid-log phase growth by rotary shaking at 150 rpm at 30°C in the dark, after which they were transferred to 150 mL of identical fresh media each in separate 500-mL size conical flasks and growth to mid-log phase was repeated. Upon reaching mid-log phase, cells that were exposed to acrolein, 10 mmol/L, and unexposed cells were extracted immediately for total DNA and this is referred to as *T* = 0. Total DNA was also extracted after 8, 30, and 120 min (*n* = 3 each exposed and unexposed cells) depending upon the experiment. To extract DNA, 20 mL of cells were aseptically removed and mixed with an equal volume of SBM, centrifuged at 4800*g* for 8 min at 4°C, and followed by two more washing steps using 40 mL of 5 mmol/L phosphate buffer washing solution (pH 7.0) with the same centrifugation conditions. DNA extractions of pelleted cells were conducted by using the PowerMicrobial Maxi DNA Isolation Kit (MoBio Laboratories, Inc, Carlsbad, CA). DNA quantification and purity was measured by absorbance at 260 and 280 nm via a Nanodrop instrument (Thermo Scientific, Wilmington, DE). Absorbance (OD_620_) of strain KK22 cells at mid-log phase was equal to ∼0.9 (V-530 model UV-visible spectrophotometer; Jasco, Tokyo, Japan).

### DNA purification and hydrolysis

Based upon the amount of DNA recovered from each sample, aliquots of solution that contained 100 *μ*g of DNA were transferred to 1.5-mL size Eppendorf tubes and Milli-Q water was removed by vacuum centrifugation (Tomy Seiko Co., Tokyo, Japan). Afterwards, bacterial DNA was enzymatically hydrolyzed to their corresponding 2′-deoxyribonucleoside-3′-monophosphates by the addition of 54 *μ*L MN/SPD in mixed buffer (17 mmol/L sodium succinate and 8 mmol/L calcium chloride, pH 6.0) that contained 67.5 units of MN and 0.225 units of SPD. Solutions were gently mixed by hand and incubated for 3 h at 37°C in a heating block (DTU-1B; Taitec, Saitama, Japan) after which 3 units of alkaline phosphatase, 30 *μ*L of 0.5 mol/L Tris-HCl (pH 8.5), 15 *μ*L of 20 mmol/L zinc sulfate, and 101 *μ*L of Milli-Q water were added. The mixture was again gently mixed and incubated for another 3 h at 37°C. Following the second digestion incubation, the mixture was subjected to vacuum centrifugation to a final volume of ∼20 *μ*L after which 100 *μ*L of methanol were added to precipitate proteins and extract DNA nucleosides. Methanol extract supernatants were transferred to new Eppendorf tubes and precipitate was extracted again with 100 *μ*L of methanol. After centrifugation, the supernatant extracts were combined in a total volume of 200 *μ*L of methanol. Lastly, methanol was removed by centrifugal concentration and the remaining 2′-deoxynucleosides were redissolved in 200 *μ*L of 30% DMSO (w/v) in Milli-Q water that contained 2′,3′-dideoxyinosine as an internal standard.

### Instrumentation

Analyses of bacterial 2′-deoxyribonucleosides were conducted by liquid chromatography electrospray ionization-tandem mass spectrometry in positive ionization mode (LC/ESI(+)-MS/MS) by using a system that consisted of a Waters 2690 Separations Module (Waters, Corp., Milford, MA) that was interfaced with a Quattro *Ultima* triple stage quadrupole mass spectrometer (Waters-Micromass, Manchester, U.K.) in-line with a Waters 2998 photodiode array detector. An aliquot of sample, 30 *μ*L, was introduced via autoinjector to a Shim-pack XR-ODS column (3.0 × 75 mm, Shimadzu, Kyoto, Japan) that was in-line with a Security Guard Cartridge System pre-column fitted with a widepore C18 cartridge (Phenomenex, Torrance, CA). The samples were eluted at a flow rate of 0.2 mL/min via a gradient that began at a ratio of 5% methanol and 95% water and which changed to a ratio of 80% methanol and 20% water over a period of 25 min. The 80:20 conditions were held for 5 min and then returned to the original starting conditions over 10 min. The sample components were delivered to the mass spectrometer by electrospray which utilized nitrogen gas as the nebulizing gas. The ion source temperature was 130°C, the desolvation temperature was 350°C, and the cone voltage was operated at 35 V. Nitrogen gas was also used as the desolvation gas, 600 L/h, and cone gas, 60 L/h, and argon gas was used as the collision cell gas at a collision cell pressure of ∼3.5 × 10^−3^ m Bar. Positive ions were acquired in SRM mode and the detection strategy was designed to detect the neutral loss of 2′-deoxyribose from positively ionized 2′-deoxynucleoside adducts by monitoring the samples transmitting their [M + H]^+^ > [M + H − 116]^+^ transitions. For each sample of bacterial 2′-deoxyribonucleosides analyzed, 100 transitions were monitored over the [M + H]^+^ range from transition 250 > 134 to transition 350 > 234. For each sample application, 32 channels were monitored simultaneously with one channel reserved for each injection to monitor 2′,3′-dideoxyinosine at transition 236.9 > 136.8. Transitions that corresponded to normal 2′-deoxynucleosides, such as 2′-deoxyadenosine, 252 > 136, and 2′-deoxyguanosine, 268 > 152, were not monitored. 2′-Deoxynucleoside abundances were evaluated by UV detection at 254 nm. Product ion scan analyses were conducted under similar conditions and the collision cell energies that were used for analyses discussed in this report were: [M + H]^+^ = 264, 266, 10 eV; 276, 25 eV; 282, 284, 8 eV; 312, 8 and 20 eV; 324, 346, 20 eV.

### DNA adductome analysis

Data from LC/ESI(+)-MS/MS analyses were processed by using MassLynx software and DNA adductome maps were produced as described previously (Kanaly et al. 2006; Chou et al. 2010). Based upon the results of adductome maps, putative DNA adducts were chosen for further investigation by product ion scan analyses using collision induced dissociation (CID). CID fragmentation pattern analyses results were compared with reviews of the literature to aid in the annotation of putative DNA adducts revealed by DNA adductomics. To conduct relative abundance analyses on DNA adductome data sets, a suite of SRM transitions were selected based upon whether a specific putative DNA adduct appeared in all three samples from at least one of the two conditions described above (i.e., cells exposed to acrolein or cells not exposed to acrolein). Results for 17 putative DNA adducts that met the criteria were manually aligned by matching putative DNA adduct peak retention time ranges with the corresponding putative DNA adduct peak areas for a specific transition, organized in tabular form and statistical analyses of the mean relative abundances of these putative DNA adducts was conducted by Student *t*-test.

### Quantification of 8-hydroxyPdG

Experimental conditions were identical to those described for DNA adductome analyses except that the SRM transition 324 > 108 was utilized. The amount of 8-hydroxyPdG in each digested DNA sample was quantified by calculating the peak area adjusted for by the recovery of internal standard, 2′,3′-dideoxyinosine (SRM transition 236.9 > 136.8) and the amount of DNA 2′-deoxynucleosides that were applied to the column. A calibration curve was obtained by using an authentic standard of 8-hydroxyPdG and the numbers of 8-hydroxyPdG adducts per 10^9^ bases in each sample were calculated based upon the ratio of guanine bases relative to total base number in the DNA of *Sphingobium* sp. strain KK22 (Maeda et al. 2013).

## Results

### DNA adductome maps

**Figure 1:**
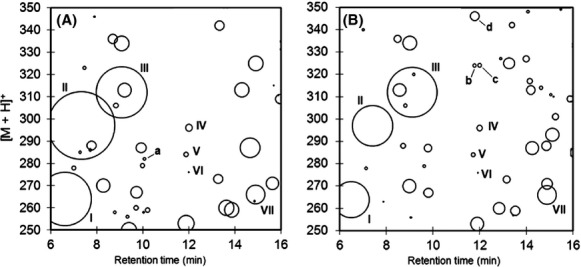
Adductome analyses of bacterial cells growing on glucose in mid-log phase (A), and bacterial cells growing on glucose in mid-log phase 30 min after exposure to acrolein (B). Putative DNA adducts discussed in the text are labeled I through VII and a through d.

Results of DNA adductome map construction for bacterial DNA after 30 min of incubation on glucose (Fig.1A) and under the same conditions plus acrolein (Fig.1B) revealed that numerous putative DNA adducts were detected in both samples over the entire range corresponding to SRM transitions 250–350. Maps showed that many of the putative DNA adducts occurred similarly under both conditions (marked with Roman numerals in Fig.1) and sometimes with similar relative abundances, such as in the cases of putative DNA adducts IV, V, and VI for example (Fig.1A and B). DNA adductome analysis also revealed that under both conditions, the most abundantly detected putative DNA adducts occurred identically and with similar area response values (putative DNA adducts I, II, III, and VII). Previous results from DNA adductome analyses indicated that the greatest numbers of putative DNA adducts were detected in a similar range, from *m/z* 250 to *m/z* 350, and this range was selected for experiments herein (Kanaly et al. 2006, 2007).

Differences between the two incubation conditions were also revealed and indicated that in the case of exposed cells, more putative DNA adducts appeared that corresponded to protonated molecules with *m/z* values greater than 280 between 11 and 16 min. In many cases different putative DNA adducts appeared under one condition that did not appear in the other condition and these are marked by lower case letters in Figure1. Among these, putative DNA adduct a, in unexposed cells (Fig.1A) and putative DNA adducts b, c and d in exposed cells (Fig.1B) are discussed in the following sections.

### Relative abundance analysis by DNA adductomics

After consideration of these results, DNA adductome analyses were conducted in triplicates to compare the relative abundances of specific putative DNA adducts that were revealed in Figure1. The results are given in Table1. Comparisons of relative putative DNA adduct abundance under the two conditions showed that overall, exposed cells resulted in greater average relative abundances. At the same time however, there also appeared to be greater variability among the relative abundances detected from exposed cells. The four most abundantly detected putative DNA adducts in this analysis, [M + H]^+^ = 266, 312, 297, and 264, average retention times equal to 14.8, 8.5, 6.2, and 8.0 min, respectively, were found to be identical to those revealed in the adductome maps in Figure1 that are labeled as I ([M + H]^+^ = 264), II ([M + H]^+^ = 297), III ([M + H]^+^ = 312) and VII ([M + H]^+^ = 266). Also in agreement with the results in Figure1, putative DNA adducts c and d which corresponded to [M + H]^+^ = 324, r/t = 12.0 min, and [M + H]^+^ = 346, r/t = 11.9 min were detected in triplicate in exposed cells and were not detected in unexposed cells (Table1). In one example case, putative DNA adducts which corresponded to [M + H]^+^ = 276, r/t = 11.9 min, and are denoted as VI in Figure1, were detected in significantly greater relative abundance in unexposed cells. Overall, retention time matching of putative DNA adducts was generally within a range of 0.1–0.2 min however wider ranges occurred in some cases. This highlights one of the challenges of conducting manual adductome peak annotation where large peaks and peaks with less than ideal shapes for example may contribute to differences in retention time values (Table1).

**Table 1:** Comparison of area response values of putative DNA adducts aligned by retention time ranges and [M + H]^+^ values

[M + H]^+^	Unexposed cells		Exposed cells	
	Area response^1^	Retention time (min)^2^	Area response	Retention time (min)
264	574 ± 309	8.0 ± 0.1	764 ± 234	7.9 ± 0.2
266	74 ± 14	14.8 ± < 0.1	177 ± 70	14.7 ± < 0.1
270^3^	51 ± 3	8.4 ± 0.3	103 ± 8	8.5 ± 0.1
276^4^	6 ± 1	11.9 ± 0.1	3 ± 4	11.8 ± 0.1
279	13 ± 3	9.9 ± < 0.1	18 ± 5	9.9 ± 0.2
284	18 ± 3	11.9 ± < 0.1	22 ± 13	11.9 ± 0.2
287^4^	29 ± 2	10.0 ± < 0.1	53 ± 15	9.8 ± 0.1
288	31 ± 11	8.5 ± 0.1	20 ± 14	8.7 ± 0.1
288^3^	16 ± 5	14.8 ± < 0.1	29 ± 2	14.7 ± 0.1
296	29 ± 15	12.0 ± 0.2	29 ± 17	11.7 ± 0.1
297^4^	1046 ± 180	6.6 ± 0.2	3223 ± 1359	5.8 ± 0.5
306	12 ± 6	8.7 ± 0.2	53 ± 39	8.3 ± 0.2
312^4^	284 ± 57	8.3 ± 0.3	1698 ± 719	8.8 ± 0.3
324	n.d.^5^	n.d.	23 ± 6	12.0 ± 0.1
334	160 ± 8	8.8 ± 0.4	86 ± 98	8.5 ± 0.5
336	160 ± 8	8.7 ± 0.2	128 ± 152	8.8 ± 0.1
346	n.d.^5^	n.d.	135 ± 156	11.9 ± 0.1

1 The normalized average area response values for each putative DNA adduct indicated plus the standard deviation. Values represent the results of separate DNA adductome analyses that were conducted in triplicate each for unexposed and exposed cells.

2 Average retention time of each putative DNA adduct based upon peak height maxima plus the standard deviation.

3 Average area response values were significantly different, *P* < 0.05 (Student *t*-test).

4 Average area response values were significantly different, *P* < 0.10 (Student *t*-test).

5 n.d.: not detected. Putative DNA adducts that corresponded to [M + H]^+^ = 324 and 346 were detected in triplicate in exposed cells but not in unexposed cells.

### Investigation of putative DNA adducts revealed by adductome analyses

Putative DNA adducts IV, V, and VI, [M + H]^+^ = 296, 284, and 276, respectively, occurred at transitions that are known to represent commonly detected forms of DNA damage that arise from oxidative stress. Indeed, three of such DNA adducts, *N*^*2*^-ethylguanine, 8-oxoguanine, and 1,*N*^6^-ethenoadenine, were identified as their 2′-deoxynucleosides in adductome analyses of DNA from human tissues and they occurred at these identical transitions (Kanaly et al. 2006, 2007; Chou et al. 2010; Matsuda et al. 2013). Because putative DNA adducts IV, V, and VI occurred at these transitions, authentic standards of *N*^*2*^-ethyl-2′-deoxyguanosine, 8-hydroxy-2′-deoxyguanosine, and 1,*N*^6^-etheno-2′-deoxyadenosine were chosen and analyzed in this study under identical LC/ESI-MS/MS conditions and their retention times were found to be 14.9, 10.4, and 13.4 min respectively (data not shown). When compared to putative DNA adducts that occurred in this bacterium at identical transitions in Table1, results indicated that putative DNA adducts IV through VI revealed by adductome analyses were not *N*^*2*^-ethylguanine, 8-oxoguanine, or 1,*N*^6^-ethenoadenine.

DNA adductomics is a top down approach and to derive further understanding from the results of analyses, targeting of unknown putative DNA adducts revealed by adductome maps for evaluation by product ion scanning may provide useful information to support identification. Specifically, when unknown adducts cannot be identified by comparison with authentic standards, product ion scan analyses that yield fragments other than the expected [M + 2H − dR]^+^ fragment may allow for scoring a putative DNA adduct as a candidate for further investigation. Considering this, product ion scan analyses were conducted on putative adducts IV through VI. Results from analyses of IV, [M + H]^+^ = 296, revealed strong product fragments at *m/z* 180 which corresponded to [M + 2H − dR]^+^ but also product fragment *m/z* 137 (Fig. S1). During product ion scan analyses of 2′-deoxynucleosides in positive ionization mode, the detection of fragments *m/z* 136 or *m/z* 152 provides evidence for 2′-deoxyadenosine and 2′-deoxyguanosine adducts because these fragments represent protonated adenine and guanine bases respectively (Hua et al. 2001; Gamboa da Costa et al. 2003). However, a product ion produced with a value of *m/z* 137 is known to occur in the fragmentation of inosines (Kambara 1982; Polson et al. 1991; Selzer and Elfarra 1996; Li et al. 2009) which provided evidence for a hypoxanthine base adduct in DNA brought about by *N*1 position alkylation of adenine and subsequent hydrolytic deamination or a Dimroth rearrangement for example (Fujii et al. 1990; Kanuri et al. 2002). Finally, among known DNA adducts that have molar masses of 295 g mol^−1^, a product ion at *m/z* 137 occurs during fragmentation of *N*^2^,*N*^2^-dimethyl-2′-deoxyguanosine (Dudley et al. 2004; Beach and Gabryelski 2011) but not its *N*^2^,*O*^6^-dimethylated isomer (Farmer et al. 1973; Fig. S1). Based upon these results, putative adduct IV was annotated for future evaluation that shall require the synthesis of authentic standards but which is beyond the scope of this report. A summary of results of putative DNA adducts revealed in adductome maps that were investigated in this study are indicated in Table2 and includes putative DNA adducts discussed in the proceeding sections.

**Table 2:** Putative DNA adducts from a bacterium revealed by DNA adductomics discussed in this study

Putative DNA adduct	[M + H]^+^	Average retention time (min)	Description
I	264	8.0	Determined to be the sodiated adduct of 5-methyl-cytidine. Detected in high relative abundance under both conditions
II	297	6.2	Identity unknown. Detected in high relative abundance under both conditions
III	312	11.8	Annotated for further evaluation as 2-hydroxyethylguanine. Six times greater relative abundance in exposed cells
IV	296	12.0	Detected under both conditions and determined not to be *N*^2^-ethylguanine. Annotated for further evaluation as hypoxanthine or *N*^2^,*N*^2^-dimethylguanine adduct
V	284	11.8	Detected under both conditions and determined not to be 8-hydroxyguanine
VI	276	12.0	Detected under both conditions and determined not to be 1,*N*^6^-ethenoadenine
VII	266	14.7	Identity confirmed as *N*^6^-methyladenine. Two times greater relative abundance in exposed cells
a	282	9.9	Annotated as a possible methylguanine adduct. Only occurred in unexposed cells
b	324	11.7	Confirmed as 6-hydroxypropanoguanine. Only occurred in exposed cells
c	324	12.0	Confirmed as 8-hydroxypropanoguanine. Only occurred in exposed cells
d	346	11.9	Annotated as a sodiated adduct of putative DNA adduct c or a possible butanone-ethenoadenine adduct. Only occurred in exposed cells

Product ion scan analysis of putative adduct V, [M + H]^+^ = 284, yielded fragments that supported that it was a 2′-deoxynucleoside adduct in that strong ions were detected at *m/z* 168, [M + 2H − dR]^+^, and *m/z* 117 which represented 2′-deoxyribose (Hua et al. 2001; Beland et al. 2005). Including detection of *m/z* 151, this spectrum was largely similar to the mass spectrum of authentic 8-hydroxy-2′-deoxyguanosine (Fig. S2). The results of analysis of putative adduct VI, [M + H]^+^ = 276, represented what is more commonly revealed in that only the [M + 2H − dR]^+^ fragment was detected; *m/z* 160.

**Figure 2:**
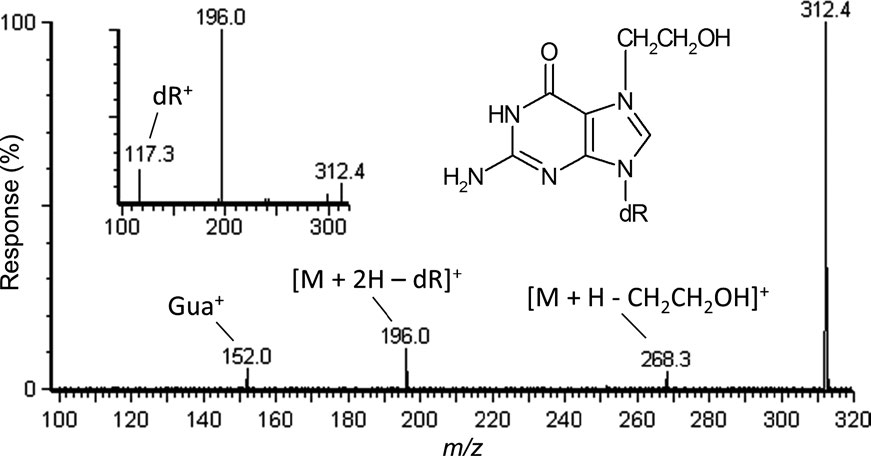
LC/ESI(+)-MS/MS product ion scan analyses of putative DNA adduct III (10 eV) which corresponded to the protonated molecule [M + H]^+^ = 312 and which was revealed by DNA adductome analysis in Figure1. Inset, results of CID fragmentation at 20 eV allowed for the detection of the *m/z* 117 deoxyribose diagnostic fragment. LC/ESI (+)-MS/MS, liquid chromatography electrospray ionization-tandem mass spectrometry in positive ionization mode; CID, collision induced dissociation.

By using approaches similar to above, analyses of the relatively abundantly detected putative adducts I through III and VII were conducted. Based upon retention time and the value of the protonated molecule, [M + H]^+^ = 264, putative adduct I was assigned to be the sodiated adduct of 5-methyl-2′-deoxycytidine following MS2 analyses of an authentic standard of this compound. Results for putative adduct II, [M + H]^+^ = 297 were inconclusive, however putative adducts III and VII yielded valuable diagnostic fragmentation ions. As shown in Figure2, product ion analyses of putative adduct III, [M + H]^+^ = 312, revealed fragments at *m/z* 196 ([M + 2H − dR]^+^) and *m/z* 152 (protonated guanine) which supported that this was a 2′-deoxyguanosine compound but also indicted a loss of 44 amu from the protonated base molecule. At the same time, a product ion at *m/z* 268 was detected and represented a loss of 44 amu from the parent protonated molecule. Taken together, these fragments provided evidence that putative adduct III was a hydroxyethylated guanine adduct as shown in Figure2. Hydroxyethyl-2′-deoxyguanosines have molar masses of 311 g mol^−1^ and correspond to [M + H]^+^ = 312 as detected for putative adduct III. It has been established in previous work that positive ionization mass analyses of *N*^7^-(2-hydroxyethyl)guanine yielded fragments identical to those detected here, *m/z* 196 and *m/z* 152, with the loss of 44 amu corresponding to loss of the hydroxyethyl group (Wu et al. 1999; Tompkins et al. 2008) but that analyses of 8-(1-hydroxyethyl)- and 8-(2-hydroxyethyl)guanine did not (Nakao and Augusto 1998). When putative adduct III was fragmented at a collision cell energy of 20 eV, further supportive evidence was provided for by the detection of the 2′-deoxyribose fragment ion at *m/z* 117 (Fig.2, inset). *N*^7^-(2-hydroxyethyl)guanine is a type of DNA damage that occurs due to oxidative stress and it is considered to be the most or second-most prevalent endogenous DNA adduct in cells (Swenberg et al. 2007; Nakamura et al. 2014). Only apurinic/apyrimidinic sites are considered to occur more frequently (Nakamura et al. 2014). In the quantitative analyses, even with the higher variability of the acrolein-exposed cells, putative adduct III was detected in approximately six times greater average abundance in exposed cells compared to unexposed cells which was significantly different (Table1). Hydroxyethylation at other positions of guanine also occur and the identity of putative adduct III shall need to be confirmed after the synthesis and analysis of authentic standards.

**Figure 3:**
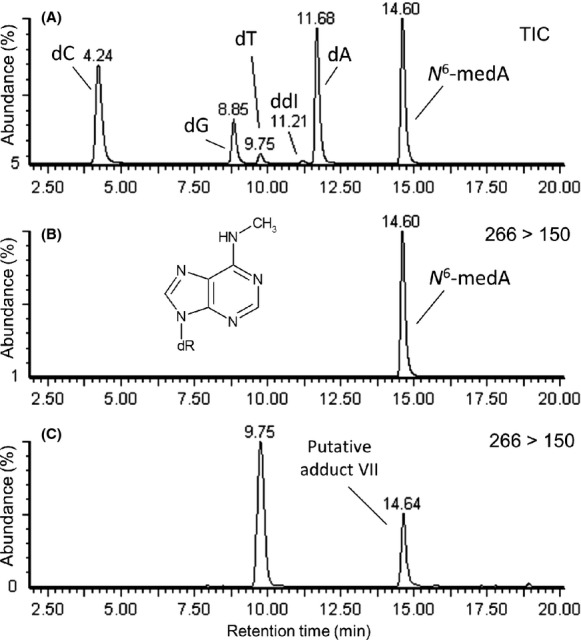
(A) LC separation of a mixture of authentic standards of 2′-deoxynucleosides 2′-deoxycytidine, 2′-deoxythymidine, 2′-deoxyguanosine, 2′-deoxyadenosine and *N*^6^-methyl-2′-deoxyadenosine plus dideoxyinosine (dC, dG, dT, dA, *N*^6^-medA and ddI respectively). (B) *N*^6^-methyl-2′-deoxyadenosine detected by LC/ESI(+)-MS/MS SRM mode at transition 266 > 150, and (C) putative DNA adduct VII detected by LC/ESI(+)-MS/MS SRM mode at transition 266 > 150. The peak that was detected at 9.75 min occurred due to the abundantly detected sodiated ion of 2′-deoxythymidine which was detected at SRM transition 265 > 149. LC/ESI (+)-MS/MS, liquid chromatography electrospray ionization-tandem mass spectrometry in positive ionization mode; SRM, single reaction monitoring.

Putative adduct VII, [M + H]^+^ = 266, yielded fragments consistent with a methylated-adenine adduct. It was identified as *N*^6^-methyl-2′-deoxyadenosine after analysis of an authentic standard. Results of analysis of 2′-deoxynucleoside authentic standards under conditions used in this study are shown in Figure3A and for the detection of the authentic standard of *N*^6^-methyl-2′-deoxyadenosine at SRM transition 266 > 150, Figure3B. The results of SRM analysis of transition 266 > 150 from the DNA of bacterial cells that were growing on glucose is given in Figure3C. Through relative abundance analyses, *N*^6^-methyl-2′-deoxyadenosine occurred on average at more than two times greater abundance in exposed cells when compared to unexposed cells but greater variability occurred in exposed cells (Table1). *N*^6^-methyladenine may be utilized by some Proteobacteria in DNA repair and/or replication and research is ongoing in regard to its role in the epigenetics of bacteria (Wion and Casadesús 2006; Kamat et al. 2011; Bendall et al. 2013). In regard to DNA methylation, product ion scan analysis of putative adduct a, which corresponded to [M + H]^+^ = 282 and was only detected in cells growing on glucose, revealed weak fragmentation ions at *m/z* 166, [M + 2H − dR]^+^ and *m/z* 152 [Gua^+^] which were representative of a methylated guanine adduct, 281 g mol^−1^.

**Figure 4:**
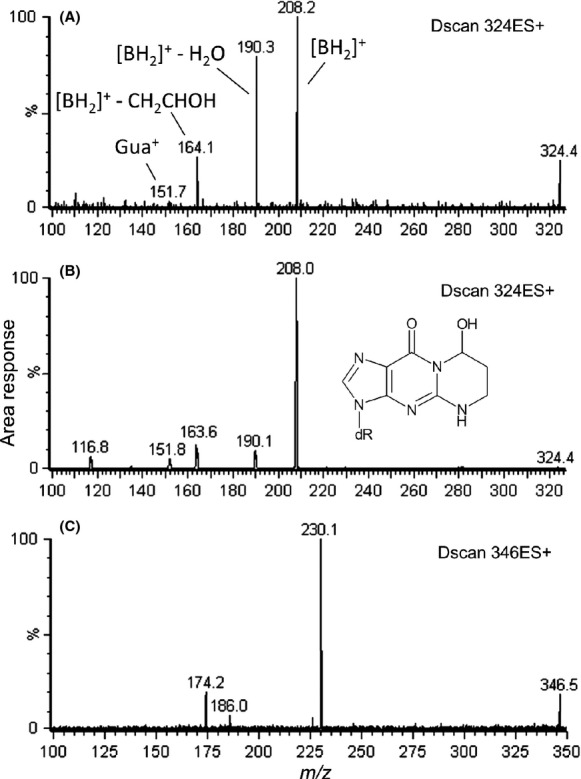
Results of LC/ESI(+)-MS/MS product ion scan analyses of (A) putative DNA adduct c, which corresponded to the protonated molecule [M + H]^+^ = 324, (B) an authentic standard of 8-hydroxy-PdG, and (C) putative DNA adduct d, which corresponded to the protonated molecule [M + H]^+^ = 346. Analyses were conducted at a CID of 20 eV. [BH_2_]^+^ is used to represent the doubly protonated base ion, [M + 2H − dR]^+^. LC/ESI (+)-MS/MS, liquid chromatography electrospray ionisation-tandem mass spectrometry in positive ionisation mode; CID, collision induced dissociation.

Putative adducts b, c, and d which corresponded to [M + H]^+^ = 324, 324 and 346 at retention times of 11.7, 12.0, and 11.9 min, respectively, were detected in exposed cells but not unexposed cells (Fig.1). Product ion scan analyses of putative adducts b and c showed similar fragmentation data and results from putative adduct c are shown in Figure4A. Based upon the results of the CID analyses and information from the literature (Cheng et al. 2003) we were led to believe that these adducts were 3-(2′-deoxyribosyl)-5,6,7,8-tetrahydro-8-hydroxy-pyrimido[1,2-*a*]purine-(3*H*)-one (8-hydroxy- PdG), and/or two stereoisomers of 3-(2′-deoxyribosyl)-5,6,7,8-tetrahydro- 6-hydroxypyrimido- [1,2-*a*]purine-(3*H*)-one (6-hydroxy-PdG). Authentic standards of these compounds were therefore synthesized and analyzed under identical conditions. Results of product ion scan analyses of these standards supported that the identities of putative adducts b and c were 6-hydroxy-PdG and 8-hydroxy-PdG, respectively, whereby four product ion fragments that were identical to the product ion fragments of putative DNA adducts b and c were revealed with *m/z* values of 208, [M + 2H − dR]^+^, 190, [M + 2H − dR − H_2_O]^+^, 164 [M + 2H − dR − CH_2_CHOH]^+^, and 152 (Gua^+^) (Fig.4A and B).

Product ion analyses of putative adduct d, [M + H]^+^ = 346, revealed ions at *m/z* 230, *m/z* 186 and *m/z* 174. Although these results may be indicative of a sodiated adduct of putative adduct c, [M + Na]^+^, results of fragmentation analyses did not confirm a loss of 23 Da from the parent protonated molecule or from the protonated base molecule. Analysis of standards of 6- and 8-hydroxy-PdGs by using SRM mode also did not reveal sodiated ions at *m/z* 346 > 230 (data not shown). Among known DNA adducts that may correspond to [M + H]^+^ = 346, the 2′-deoxynucleoside of butanone-ethenoadenine is a lipid peroxidation-derived lesion that has been detected in vivo and has a molar mass of 345 g mol^−1^ (Kawai et al. 2010). Because the retention time of putative adduct d was nearly the same as the adduct identified as 8-hydroxy-PdG (Table2) it was annotated as either a sodiated adduct of 8-hydroxy-PdG or as a possible butanone-ethenoadenine adduct. Further investigation shall be required.

### Time course analyses and absolute quantification of hydroxy-PdG adducts in strain KK22

**Figure 5:**
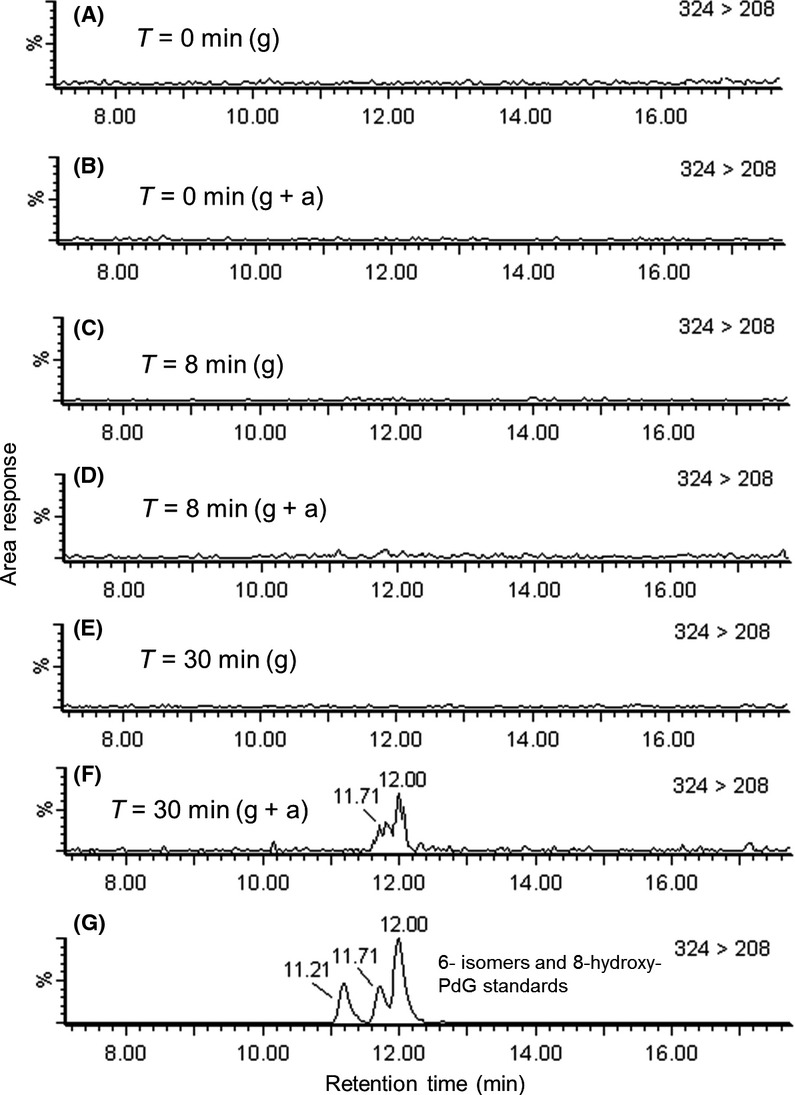
Results of LC/ESI(+)-MS/MS SRM mode time course analyses of DNA-derived 2′-deoxynucleosides extracted from a soil bacterium. SRM transition 324 > 208 was monitored in samples taken at *T* = 0 (A and B), *T* = 8 (C and D) and *T* = 30 min (E and F) from cells growing on glucose (g) and from cells growing on glucose after exposure to acrolein (g + a). The signal to noise (S/N) ratios for putative DNA adduct peaks at 11.7 and 12.0 min in (F) were six and 10 respectively. The results of analysis of three authentic standards of 6-, 6-, and 8-hydroxy-PdG which eluted at retention times of 11.2, 11.7, and 12.0 min, respectively, are given in (G). LC/ESI (+)-MS/MS, liquid chromatography electrospray ionization-tandem mass spectrometry in positive ionization mode; SRM, single reaction monitoring.

**Figure 6:**
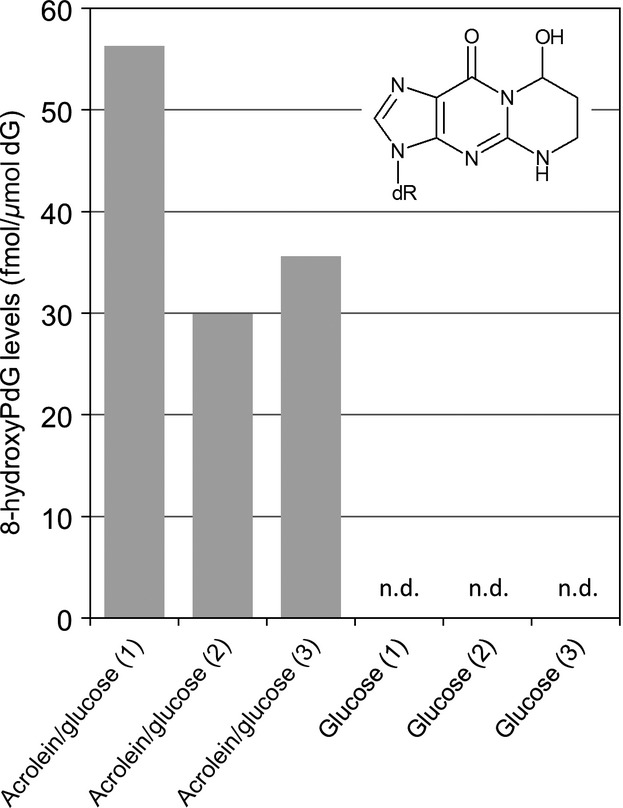
Results of quantification of 8-hydroxy-PdG in the DNA of bacterial cells that were exposed or not exposed to acrolein.

The results of time course analyses of bacterial cells that were grown on glucose and exposed or not exposed to acrolein (Fig.5) confirmed that putative DNA adducts b and c as revealed in the adductome map for acrolein-exposed cells in Figure1B were 6-hydroxy-PdG and 8-hydroxy-PdG. At the same time, time course analyses revealed that these hydroxy-PdG adducts both appeared in bacterial DNA as rapidly as 30 min after exposure to acrolein but not before 8 min (Fig.6). When cells were grown in triplicate for 120 min during exposure to acrolein, 8-hydroxy-PdG was detected in all cases, average retention time = 12.0 min, but 6-hydroxy-PdG was not, which pointed toward the transience of these adducts. The results of absolute quantitation of 8-hydroxy-PdG in bacterial DNA are given in Figure6 whereby levels in exposed cells ranged from 30 to 56 fmoles per *μ*mole of 2′-deoxyguanosine and corresponded to between 9.7 and 18.1 adducts per 10^9^ bases. In cells that were not exposed to acrolein, 8-hydroxy-PdG and 6-hydroxy-PdGs were not detected. Based upon the detection limits for 6-hydroxy- and 8-hydroxy-PdGs, the numbers of 8-hydroxy-PdG adducts in unexposed cells were less than 0.5 adducts per 10^8^ bases and the numbers of 6-hydroxy-PdG adducts were less than 1.9 adducts per 10^8^ bases.

## Discussion

Sphingomonad bacteria survive and may thrive in soil environments that are contaminated with genotoxic hydrocarbon pollutants released at petroleum exploration and processing sites, including hydraulic fracturing sites which also utilize acrolein as a biocidal agent. Toward the development of methods to investigate DNA modifications in microorganisms, a soil *Sphingobium* strain was investigated by DNA adductomics. It was hypothesized that application of DNA adductomics would allow for the detection of various unknown DNA adducts and that some might be identified as specifically occurring due to exposure to acrolein. It was also hypothesized that DNA adducts due to oxidative stress and lipid peroxidation might be detected in exposed cells when compared to unexposed cells. The results of these experiments supported the first hypothesis but not the second.

DNA adductome analyses revealed numerous putative adducts under both conditions including some that appeared to occur only in exposed cells and these results confirmed initial results presented in Figure1. The putative DNA adducts, b, c, and d (Fig.1), which were specific to cells that were exposed to acrolein, corresponded to molar masses of 323 (r/t = 11.7), 323 (r/t = 12.0 min), and 345 (r/t = 11.9 min) g mol^−1^ respectively. Results of past in vitro and some in vivo studies have shown that there are many possible DNA adducts caused by direct alkylation reactions with acrolein and that all four of the main DNA bases, guanine, thymine, adenine, and cytidine are susceptible (Chung et al. 1984; Marinelli et al. 1990; Chenna and Iden 1993; Nechev et al. 2000; Cheng et al. 2003; Pawłowicz and Kronberg 2008). In the case of putative DNA adducts b and c described herein, product ion scan analyses revealed the presence of *m/z* 152 which supported that they were guanine adducts. Based upon this information and the predicted molar masses for putative DNA adducts b and c, hydroxyPdG cyclopropano-guanine authentic standards were analyzed and the identities of b and c were confirmed to be acrolein exposure-derived adducts of guanine, 6- and 8-hydroxy-PdG; 6-hydroxy-PdG had not been detected before in a prokaryote. Other known adducts of acrolein DNA alkylation that fell within the range of molar masses analyzed in this study, such as cyclopropano-adenine adducts, were not detected (Nechev et al. 2000; Pawłowicz et al. 2006). Interestingly, 8-hydroxyPdG is considered to be a good substrate for nucleotide excision repair (NER) in *E. coli* and through NER plus daughter strand gap repair, it was reported that cells were relatively protected (Yang et al. 2001). On the other hand, 6-hydroxyPdG was reported to be more genotoxic than 8-hydroxyPdG because it strongly blocked DNA synthesis (Yang et al. 2002). In the work herein, 6-hydroxyPdG was detected after 30 min but not after 2 h and it may have been that cells that incurred 6-hydroxyPdG adducts expired rapidly.

Absolute quantification of 8-hydroxyPdG in triplicate adductome experiments showed that adduct levels were in the range of ∼10–20 lesions per 10^9^ nucleotides and these levels were similar to levels of 8-hydroxyPdG detected in human tissues (Zhang et al. 2007; Chou et al. 2010). On a moles of adduct per mole guanine basis, 8-hydroxyPdG adducts detected in this bacterium (average, 41 fmoles/*μ*mole) were greater than background levels in human leukocytes (3–25 fmoles/*μ*mole; Nath et al. 1996), or were similar to or were ∼5- to 10-fold less than 8-hydroxyPdG adducts detected in unexposed rat tissues in different studies (Nath et al. 1996, 1998; Chung et al. 1999). When *Salmonella typhimurium* strains were exposed to 10 mmol/L acrolein, 8-hydroxyPdG adducts were reported from 2–4 *μ*moles/mole which is 50–100 times greater than strain KK22 (Foiles et al. 1989). These differences may be due to the increased sensitivity of these *S. typhimurium* strains to chemicals (e.g. *rfa* mutation) or due to better ability of strain KK22 to manage DNA damage for example. Because 8-hydroxyPdG adducts were not detected in unexposed cells, it may indicate that these adducts are not highly occurring endogenous adducts in bacteria when compared to rats and humans. It must also be considered that lipid peroxidation-derived toxicants such as acrolein may not be occurring in high abundance intracellularly in this strain.

At the same time, acrolein does not only produce DNA adducts by direct alkylation – it is a known initiator of both oxidative stress and lipid peroxidation. Intracellularly, acrolein is transformed into its corresponding epoxyaldehyde by H_2_O_2_, by reactions with endogenous fatty acid hydroperoxides or by enzymatic mechanisms (Chen and Chung 1996; Loureiro et al. 2004). When epoxyaldehydes react with DNA they lead to the formation of etheno adducts (Chung et al. 1996). Additionally, known lipid peroxidation products such as 4-hydroxy-2-nonenal, for example, may be caused by acrolein-induced increases in intracellular oxidative stress and may react to produce etheno adducts (Lee et al. 2005; Chen et al. 2010). Strain KK22 cells were exposed to 10 mmol/L acrolein, however, etheno adducts were not detected in all samples by DNA adductomic analyses. Also, compared to reactive free radicals, aldehydes are relatively long-lived and can diffuse in the cell to reach and attack targets which are distant from the exposure site (Cabiscol et al. 2010). Interestingly, 1,*N*^6^-etheno-2′-deoxyadenosine is generally readily detected in investigations of eukaryotic cell DNA (Chung et al. 1996; Kadlubar et al. 1998) including previous DNA adductomics studies (Kanaly et al. 2006, 2007; Chou et al. 2010; Matsuda et al. 2013). Results from the bacterial adductome analyses conducted herein therefore indicated that strain KK22 may not have been influenced by acrolein epoxyaldehydes caused by H_2_O_2_ or by fatty acid hydroperoxides and that lipid peroxidation was not a major factor for causing DNA damage in these cells. That intracellular monounsaturated fatty acid (MUFA) hydroperoxides were not present in high concentration or H_2_O_2_ was tightly controlled may account for these results. The detection of putative 2-hydroxyethylguanine adducts in this study notwithstanding due to their apparent relatively high occurrence rates in living organisms in general. Recently, acrolein and malondialdehyde were detected in bacteria when under oxidative stress which implicated that lipid peroxidation may have occurred (Maness et al. 1999; Pérez et al. 2008; Joshi et al. 2011). Similarly, Pradenas et al. (2012, 2013) reported a direct relationship between MUFA and lipid peroxide generation in *E. coli* and demonstrated that MUFAs are substrates for aldehyde generation by tellurite. Taken together, these reports posit that lipid oxidation in bacterial cells may be a part of bacterial metabolism. Compared to *E. coli* however, strain KK22 grows on high concentrations of PAHs and may have better coping mechanisms to deal with oxidative stress during aromatic hydrocarbon biotransformation and subsequent production of PAH-catechols and PAH-quinones for example (Kim et al. 2003; Kanaly and Hamamura 2013). Direct alkylation products of acrolein were detected by 30 min in exposed cells and indicated that acrolein had entered the cells. Even so, lipid peroxidation-related DNA adducts caused by oxidative stress that are commonly detected in eukaryotic cell systems such as 1,*N*^6^-etheno-2′-deoxyadenosine, [M + H]^+^ = 276, and DNA adducts of glyoxal [M + H]^+^ = 326 and crotonaldehyde, [M + H]^+^ = 338, were not detected in bacterial DNA in this study.

Relative abundance analyses showed that 15 putative DNA adducts were consistently detected in exposed and unexposed cells, that some putative DNA adducts seemed to appear only in exposed cells, and that overall, greater relative abundances were detected in exposed cells for the most abundantly detected putative adducts. Based upon relative abundances and predicted molar masses of unknown putative DNA adducts revealed in adductome maps, candidates were selected for product ion scan analyses and in some cases clear diagnostic fragmentation ions were differentiated against a stable baseline which provided further support that these were DNA adducts. Furthermore, the validity of approach was demonstrated by the unambiguous identification of three DNA adducts in bacterial DNA that were originally revealed only by the adductome mapping technique. The annotation of other putative adducts as strong candidates for further evaluation based upon their fragmentation patterns and predicted molar masses pointed toward the efficacy of directed product ion scan analyses. Because DNA adductomics is truly a top down approach, there are many challenges to working through the identification process of the numerous putative DNA adducts that are revealed by the adductome maps.

*N*^6^-methyl-2′-deoxyadenosine, the 2′-deoxynucleoside of *N*^6^-methyladenine, was identified as one of the most abundantly detected putative DNA adducts in all samples. Methylation of adenine at the *N*^6^ position in bacteria has recently become a DNA modification of interest due to its proposed effects on different cellular epigenetic control mechanisms including that it has been implicated as an epigenetic signal that may regulate physiological processes, including repair processes (Casadesús and Low 2006). In this study, *N*^6^-methyl-2′-deoxyadenosine was detected on average in more than two times greater abundance in exposed cells and whether this is related to toxic stress in these cells may be the subject of future investigation. Results of product ion scan analyses of another putative adduct that was detected in relative high abundance in all cells, which corresponded to the protonated molecule [M + H]^+^ = 312, provided evidence that it was *N*^7^-(2-hydroxyethyl)guanine. This was consistent with observations that this adduct generally occurs in high amounts in living organisms and represented one of the first detections of this adduct in vivo in a soil bacterium (Wu et al. 1999; Tompkins et al. 2008).

Using bacteria as potential indicators of toxicity in soils that were exposed to hydrocarbon pollutants was recently reported (van Dorst et al. 2014). In the past, genotoxicity evaluations have also been conducted by using microorganisms to assess the impacts of wastes (Donnelly et al. 1995). From an ecotoxicology perspective, detection in soil bacteria of DNA modifications such as methylation, or DNA damage such as Michael addition products – both as described in this report – may provide understandings in regard to environmental disturbances and/or the types of toxicants that are present in the local environment. Epigenetic studies are impacting the field of ecotoxicology whereby epigenetic changes (mainly DNA methylation) are now being reported to be induced by environmental stresses, including exposures to environmental pollutants (Vandegehuchte and Janssen 2014). DNA adductomics was applied in this study as part of the development of methods to investigate the applicability of the approach to investigating DNA modifications in soil microorganisms that may serve as markers of environmental pollution and potential future genotoxic effects in situ. At least two DNA adducts that are known to occur through exposure to acrolein were detected after acrolein exposure. Because these types of adducts occur through a specific exposure scenario, they may represent potential markers of acrolein contamination. Results of timecourse analyses revealed that 6-hydroxyPdG was not detected after 120 min however. Overall, 8-hydroxyPdG may be a potential marker of acrolein exposure, but 6-hydroxyPdG, which was detected transiently, less so. At the same time, this research contributes to the field of DNA adductomics which is under development. Future work in bacterial systems that aim to confirm the identities of putative DNA adducts revealed in this study shall provide further information in regard to DNA damage in bacteria. In future applications, utilization of DNA adductomic approaches to investigate bulky DNA adducts caused by exposure to environmental pollutants such as PAHs may be explored.

## Supporting Information

**Figure S1.** Fragmentation pattern acquired from product ion scan analysis of putative DNA adduct IV which corresponded to the protonated molecule [M + H]^+^ = 296. **Figure S2.** Fragmentation patterns acquired from product ion scan analyses of (A) putative DNA adduct V which corresponded to the protonated molecule [M + H]^+^ = 284 and (B) an authentic standard of 8-hydroxy-2′-deoxyguanosine, [M + H]^+^ = 284.
