# Supplementary material for: Application of DNA adductomics to soil bacterium Sphingobium sp. strain KK22
Source: Microbiologyopen. 2015 Aug 25;4(5):841–56. doi: 10.1002/mbo3.283 (PMC4618615; doi:10.1002/mbo3.283)
Supplement: Supplementary file 1 — Figure S1. Fragmentation pattern acquired from product ion scan analysis of putative DNA adduct IV which corresponded to the protonated molecule [M + H]+ = 296. Figure S2. Fragmentation patterns acquired from product ion scan analyses of (A) putative DNA adduct V which corresponded to the protonated molecule [M + H]+ = 284 and (B) an authentic standard of 8-hydroxy-2′-deoxyguanosine, [M + H]+ = 284. [file mbo30004-0841-sd1.docx]

**Supporting Information for:**

Application of DNA adductomics to soil bacterium *Sphingobium* sp. strain KK22


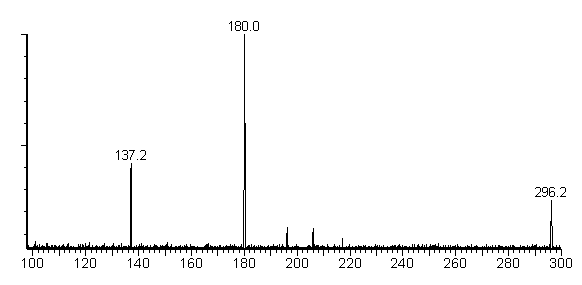


Abundance (%)

*m/z*

+

+

[M + 2H – dR]^+^

**Fig. S1** Fragmentation pattern acquired from product ion scan analysis of putative DNA adduct IV which corresponded to the protonated molecule [M + H]^+^ = 296.


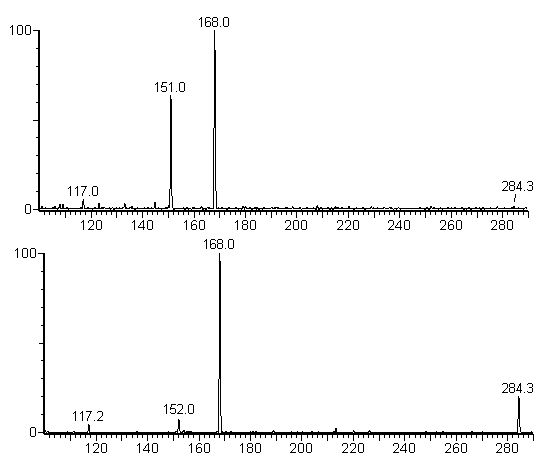


Abundance (%)

*m/z*

Abundance (%)

H

[M + 2H – dR]^+^

[M + 2H – dR]^+^

Gua^+^

dR^+^

dR^+^

A

B

**Fig. S2** Fragmentation patterns acquired from product ion scan analyses of (A) putative DNA adduct V which corresponded to the protonated molecule [M + H]^+^ = 284 and (B) an authentic standard of 8-hydroxy-2'-deoxyguanosine, [M + H]^+^ = 284.
